# State of the Art in Alcohol Sensing with 2D Materials

**DOI:** 10.1007/s40820-019-0363-0

**Published:** 2020-01-21

**Authors:** Ramin Boroujerdi, Amor Abdelkader, Richard Paul

**Affiliations:** 1 Faculty of Science and Technology, Bournemouth University, Talbot Campus, Fern Barrow, Poole, BH12 5BB UK; 2 Department of Engineering, University of Cambridge, Cambridge, CB3 0FS UK

**Keywords:** Sensors and biosensors, Alcohol probes, Electrochemical detectors, Ethanol metabolites, 2D materials

## Abstract

Since the discovery of graphene, the star among new materials, there has been a surge of attention focused on the monatomic and monomolecular sheets which can be obtained by exfoliation of layered compounds. Such materials are known as two-dimensional (2D) materials and offer enormous versatility and potential. The ultimate single atom, or molecule, thickness of the 2D materials sheets provides the highest surface to weight ratio of all the nanomaterials, which opens the door to the design of more sensitive and reliable chemical sensors. The variety of properties and the possibility of tuning the chemical and surface properties of the 2D materials increase their potential as selective sensors, targeting chemical species that were previously difficult to detect. The planar structure and the mechanical flexibility of the sheets allow new sensor designs and put 2D materials at the forefront of all the candidates for wearable applications. When developing sensors for alcohol, the response time is an essential factor for many industrial and forensic applications, particularly when it comes to hand-held devices. Here, we review recent developments in the applications of 2D materials in sensing alcohols along with a study on parameters that affect the sensing capabilities. The review also discusses the strategies used to develop the sensor along with their mechanisms of sensing and provides a critique of the current limitations of 2D materials-based alcohol sensors and an outlook for the future research required to overcome the challenges.

## Highlights

- A current review on the applications of graphene and other two-dimensional (2D) materials in alcohol detection.
- A thorough discussion on the fundamental principles and the advantages of using 2D materials in sensing alcohol.
- Critical discussion of the current limitations of alcohol sensors and the role of 2D materials in addressing the challenges.

## Graphical abstract

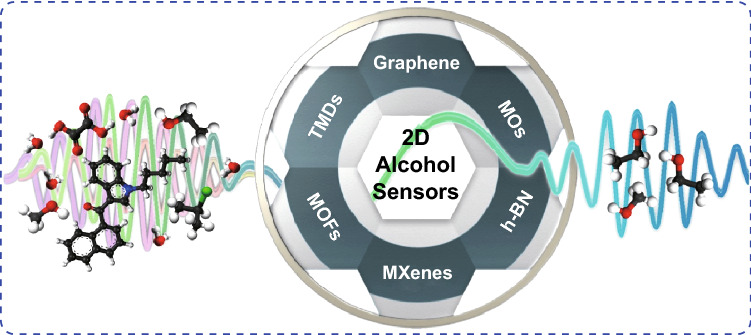

## Introduction

Developing a selective, sensitive, fast, and reliable measurement of alcohol is important in many fields including a wide range of industries, such as pulp or food industries, clinical, and forensic assays and also agricultural and environmental analysis. However, ensuring the safety and quality of food and also medical products can be complicated, and there is always a need for developing new generation sensors with more modifications [1–5]. Volatile alcohols such as ethanol are often found in workplaces and laboratories, as well as medical, pharmaceutical, and food industries, and long-term exposure to even low concentration of alcohol vapour can cause eyesight disturbance, nasal and mucous membrane inflammation, conjunctival inflammation, respiration disruption, nerve disease, lung irritation, and even death. Thus, monitoring alcohols in the air, because of their flammable nature, is essential in certain working places [6, 7]. In addition to chemical and industrial studies, adulterated alcoholic beverages present forensic interest due to possible application in a criminal activity [8].

Several methods have been used to measure different analytes in complex matrixes including chromatography, immune-chromatography, mass spectrometry, nuclear magnetic resonance, polymerase chain reaction, ultraviolet–visible spectroscopy, Fourier-transform infrared spectroscopy (FTIR) and IR, surface-enhanced Raman spectroscopy (SERS) and Raman, circular dichroism spectroscopy, spectrofluorimetric, which all are still applicable [9–20], but they have their boundaries, and some of these techniques suffer from low sensitivity and selectivity on their own. Issues of portability, slow responses, and complicated operation could also be mentioned as some of their problems [21]; thus, there is a need for new analytical procedures to provide rapid, specific analysis, and simple result interpretation [22]. Developing a new generation of nano-sized detectors to replace conventional methods is one of the possible ways to solve these issues. Nanosensors offer a range of applications, especially in the area of chemical and biological sensing, because of their large surface area, biocompatibility, and vast range of properties.

Several nanomaterials and nanostructures have been used in developing alcohol sensors. Enzyme-based sensors that change colour in response to alcohol oxidation are one example. The dip-stick sensor was developed by immobilisation of a selective enzyme over a polyaniline film and casted over a cellulose layer, so a colour change from green to blue was used as a proof of alcohol presence (> 1%) in the sample [23]. The sensor was sensitive to a range of alcohols, yet the sensitivity decreased with increasing the size of the aliphatic chain. Nanostructures based on metal oxides were also studied in the literature for alcohol sensors due to high density of functional groups on its surface that can readily react with the alcohol substances. For example, Zhu et al. fabricated sensors using Ag–TiO_2_ core–shell nanostructure and used it to sense ethanol gas at room temperature. The detection limit was reported to be as low as 0.15 ppm [24]. However, the linear range of the sensor was short, covering 0.15 to 5 ppm. Nanoporous silicon was also used to detect ethanol gas at room temperature by monitoring the changes in the resistance [25]. The detection limit of the nanoporous pillar array was reported to be 50 ppm to sense ethanol in air, yet its selectivity to other alcohol was not studied.

A recent study shows that a new hybrid carbon nanostructure made of graphene and double-walled carbon nanotube (G–DWCNT) was able to detect ethanol gas. The response time of the G–DWCNT sensor was 428 s, which is slightly faster than solo DWCNT (480 s) [26]. Some of the one-dimensional nanoparticles also offer long linear ranges, such as the SnO_2_-functionalised gallium nitride nanowires (GaN–NWs) UV-assisted sensor developed by Bajpai et al., which was able to sense various alcohols from 1 to 5000 ppm. However, the percentage response of this sensor to smaller alcohols (methanol and ethanol) was stronger than alcohols with longer aliphatic chains. Current was increased when the UV light was on and decreased when it was off; since the current was changing in the presence of carrier gas (breathing air), the current recorded with 40 sccm of air under UV was used as a reference for their study [27]. Stannum-doped mesoporous nickel oxide nanowires are another ethanol gas sensor which was able to sense ethanol in a linear range from 5 to 1000 ppm at 340 °C. The author argued that Sn doping increased the total resistance of the target gas and reported that doping increased the sensitivity up to 15.60 from 2.16 reported for pure NiO NWs [28]. The alcohol gas sensor developed from Zn(II) ions and benzene tricarboxylic acid (btc) as anionic organic linkers is an example of metal organic frameworks-based alcohol sensors, where in the presence of alcohol gases, through an exchange mechanism, alcohols will replace waters in the structure of Zn_3_(btc)_2_·12H_2_O (Zn-btc) [29]. MOF-based alcohol sensor, which was able to detect gaseous alcohol in ppm levels, was made of two zeolitic imidazolate framework materials (ZIF-8 and ZIF-93) grown on fibre optic-based surface plasmon resonance to serve as optical sensors. ZIFs have small pores offering high chemical stability which make them good candidates for sensing purposes, and they were able to sense MeOH with LoD of about 2.5 ppm (ZIF-8-SPR probe) and n-BuOH with LoD of around 73 ppm (ZIF-93-SPR probe) [30].

Optical sensing systems are also well-studied methods of sensing alcohols. Over two decades ago, fibre optics near-infrared spectroscopy was applied to measure alcohol levels [31], yet in more recent times, more sensitive methods in conjunction with novel nanoparticles have been introduced. Recently developed hybrids of fibre optics with multi-walled carbon nanotubes/Co_3_O_4_ were reported to offer high sensitivity (35 counts/ppm for ethanol and 51 counts/ppm for ammonia). The sensor also showed good selectivity in gas samples [32]. Another alcohol sensor was developed from gold-deposited surface plasmon resonance (SPR)-based glass rod sensor, which was coated with an α-mercaptoethyl-ω-methoxy polyoxyethylene (PEG thiol) layer and a Teflon AF2400 over-layer. The sensor offered good selectivity for water and ethanol [33]. Turn-on fluorescence based on terphenyl-ol derivatives is another example of optical sensors. The mixture of terphenyl-ol and sodium carbonate was found to respond selectively to ethanol by emitting a sky-blue light; it is also reported that combination of that mixture with water-absorbent polymer leads to better detection limit for sensing ethanol [34]. The optical fibre developed from Si quantum dots (QDs) was able to detect ethanol and water after 15 s exposure. The response was based on the changes in the fluorescence intensity of Si-QDs, which increases when they are exposed under blue light irradiation to alcohol and water vapours. The sensor suffers from agglomeration of the QDs on the fibres surface which leads to response variations in different samples [35].

When it comes to sensory applications, four specific properties of two-dimensional (2D) nanostructures make them suitable candidates: (a) the electron confinement in these materials without interlayer interactions, especially in monolayer structures, enables compelling electronic properties compared to other nanomaterials and makes them attractive candidates for electrochemical sensory applications. Easier flow of electrons throughout 2D structures, due to minimised interactions, leads to faster response to analytes in electrochemical sensors; (b) the atomic (or molecular) thickness offers them maximum mechanical flexibility and strength which leads to development of more enduring sensors; (c) the optical transparency, making them promising for the fabrication of highly flexible and transparent optical and electrochemical sensors, which make them favourable for developing modern wearable devices and tattooed sensors; (d) the large specific surface area allows 2D materials to interact more with the environment which makes them highly favourable for sensory applications and development of highly sensitive surfaces [36–41]. Therefore, materials with ultrathin 2D morphologies have received great interest for their possible applications in sensors and biosensors, particularly for drug and alcohol detection.

Furthermore, 2D materials could promise a range of new or optimised behaviours. As an example, ultrathin 2D nanomaterials, especially single-layer nanosheets, where interlayer interactions are absent or limited, prepare the media suitable for unique electrical properties compared to other nanomaterials [42]. They also possess exceptional properties, due to their finite bandgap, superior flexibility, absence of dangling bonds, and significant resistance to short channel effects, which all could suggest a new generation of smart electronic, optoelectronic, and energy devices [43–59], where the first two promise the modern generation of sensors and biosensors and the last one offers developed, long lasting batteries, and energy devices. In addition, 2D layered nanomaterials are advantageous for gas sensing application specifically due to their large surface area, which facilitates surface reactions [60].

## 2D Materials as Sensors and Biosensors

2D materials take in a large family of organic and inorganic compounds and encompass a wide range of elemental compositions and properties. While graphene and graphene-based materials are at the forefront of the studied sensor materials, other 2D materials are also receiving increasing attention from both the academic and industrial communities. In this section, we review the structures and properties of the most relevant 2D materials to sensors. There are far more 2D materials than will be discussed in the following sections, such as phosphorene [61], silicene [62], antimonene [63], and 2D polymers [64], but here we discuss only stable materials with sensory applications in detecting alcohols.

### Graphene and Graphene Oxide (GO)

Carbon nanotubes, fullerenes, mesoporous carbons, graphene nanosheets, and carbon dots are different types of carbonaceous nanomaterials [65–69], which are mostly studied for their sensory applications. Yet none of the carbon materials can match graphene in this area, owing to its superior surface area, high thermal, and electrical conductivity, excellent mechanical strength, and low manufacturing cost and simple synthesis. Despite the fact that many other 2D materials have been introduced after the discovery of graphene [70], graphene is still considered as the most important known 2D material, because of the mentioned characteristics.

Graphene is a layer of carbon atoms with packed hexagonal structure with *sp*^2^ carbon bonds. One of the unique properties of graphene is its working as a zero-bandgap 2D semiconductor with a tiny overlap between the valence and conduction bands. It can also exhibit a strong ambipolar electric field effect in a way that the charge carrier concentrations of up to 10^13^ cm^−2^ and room temperature mobility of approximately 10,000 cm^−2^ s^−1^ are measured [36, 70, 71]. Also, an unusual half integer quantum hall effect for both electron and hole carriers in graphene has been observed by adjusting the chemical potential using the electric field effect [72, 73]. In addition, graphene is highly transparent, with absorption of about 2.3% towards visible light [74]. Its thermal conductivity is measured with a value of 5000 W mK^−1^ for a single-layer sheet at room temperature [75]. Moreover, graphene has excellent mechanical strength and is known as a very firm material; Young’s modulus of graphene is approximately 1 Tera Pascal (TPa) [76], and it has proved to be one of the strongest materials ever measured [76, 77]. Thermal stability of graphene makes it a suitable candidate for high-temperature gas sensing, while its mechanical strength offers long-lasting sensors. In addition, its fast electron mobility means faster response towards the analyte, which is highly favourable for electrochemical sensors. High transparency of graphene turns it into a better choice for developing wearable devices and tattooed sensors.

**Fig. 1:**
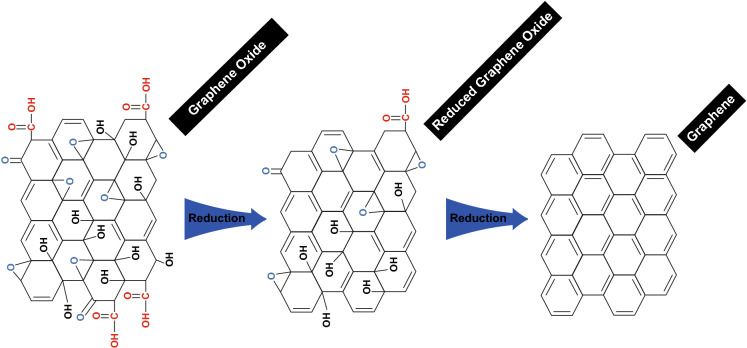
Comparing graphene oxide, reduced graphene, and highly reduced (cleared) graphene

With regard to the sensory applications, other graphene derivative materials have been used successfully. As shown in Fig. 1, 2D carbon-based nanostructures, depending on the number of oxygen groups attached to their surface, could be called graphene oxide (GO) or reduced graphene oxide (rGO) [78]. The oxygen functional groups, such as epoxides, carboxyl, hydroxyls, and alcohols, are located on the edge and surface of the sheet with a carbon to oxygen ratio of approximately 2:1 for fully oxidised GO [79].

GO is produced by subjecting graphite to a strong oxidising atmosphere. Brodie demonstrated the first synthesis method of GO by adding potassium chlorate to a slurry of graphite in fuming nitric acid [80]. Staudenmaier improved this method by adding the chlorate in small portions throughout the reaction [81]. The process is slow and involves the generation of the toxic gas(es) NO_2_, N_2_O_4_, and/or ClO_2_; the latter also being explosive [82]. Hummers and Hoffman introduced the most currently used method to prepare GO [83]. They oxidised graphite with KMnO_4_ and NaNO_3_ in concentrated H_2_SO_4_. After oxidising the bulk graphite, GO sheets are exfoliated from the oxidised bulk via sonication in aqueous solution. Several other methods have been recently suggested to produce GO based on the electrochemical oxidation in aqueous solution [84].

**Fig. 2:**
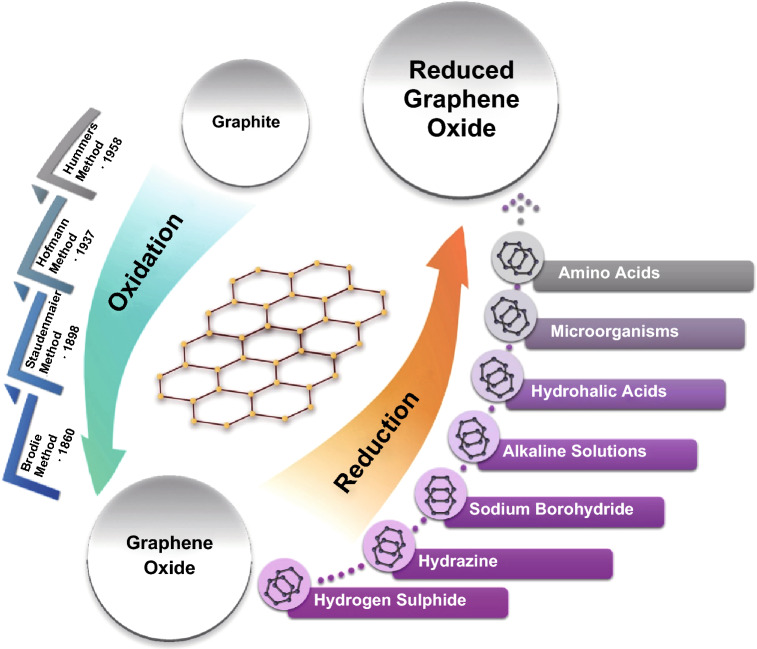
Schematic representation of some of the introduced GO and rGO production methods

Many different methods have been developed for reducing GO to recover the graphene properties. These methods can be broadly classified as chemical, thermal, electrochemical, or a mixed reduction process. Chemical processes involve the use of reducing agents such as hydrazine [85], hydrohalic acids, NaBH_4_ solutions [86], vitamin C [87], micro-organisms [88], and amino acids [89]. The chemical reduction could also be achieved with the help of electropositive elemental metals, such as iron [90], zinc [91], and aluminium [92]. The degree of reduction, as identified by the C/O ratio, depends on the reducing power of the agents. Chemical reduction methods usually suffer from high cost and may produce toxic gases or waste. Thermal reductions involve heating GO in an inert or reducing atmosphere and removal of oxygen groups in the form of carbon oxides or hydrocarbon gases. The thermal methods generally produce materials with a high C/O ratio but suffer from high defect density due to the removal of carbon atoms from the graphene basal plane to form the carbon gases [93]. Electrochemical reduction usually takes place at the cathode, either by the direct deoxidation of GO or by electrodepositing a reducing agent [84]. Some of the oxidation methods of graphite to GO and the chemical reduction of GO by some reductants are shown in Fig. 2.

Both graphene and GO could be used as sensors and biosensors, but the functionalisation of GO, because of its oxygen groups, is much easier than the reduced form. However, both forms could be used as a base material for developing a sensor. Graphene and GO have been studied for sensory application in two main areas: (a) in electrochemistry [94–97], to develop electrochemical sensors based on their conductive behaviour and (b) in spectrofluorometry [98–101], to develop fluorescent probes based on their quenching ability. Other optical methods exist, such as infrared sensors [102], porous sensors [103], and bent sensors [104], but they are rarely used.

In order to develop more sensitive and selective sensors, several methods have been suggested to alter the chemical and physico-chemical properties of graphene and GO. The term functionalised graphene is commonly used to describe chemically modified graphene. Several methods of functionalisation have been suggested including polymer coating, heteroatom doping, attaching to molecular imprinted polymers (MIP), coating with nanoparticles, and direct bonding to an organic or bio-organic molecule [105–110]. It must be mentioned that when it comes to functionalisation, graphene oxide is easier to functionalise than pristine graphene, mainly because the oxygen functional groups could work as a link to attach the foreign molecules and the defects on the GO provide active nucleation sites. One method to achieve GO functionalisation involves using the carboxylic acid groups of GO for attaching to an amine group, which enables attachment of small porphyrins to large-size structures like fullerene (C_60_) [111–113] on the graphene.

### Transition Metal Dichalcogenides (TMDs) and Transition Metal Carbides, Nitrides, and Carbonitrides (MXenes)

Layered metal chalcogenides are made of stacked planes of covalently bonded chalcogen and metal atoms, and the adjacent layers are connected together by van der Waals interactions. The ability to prepare these groups of nanosheets in high yield and large scale via various methods, such as mechanical exfoliation [114], electrochemical lithiation [115], liquid exfoliation with sonication [116], and chemical vapour deposition (CVD) growth [117], has led to increasing studies on their hybridisation with other materials to create novel functional composites, aiming to engineer their chemical, physical, and electronic properties and thus achieve good performance for some specific applications.

One of the most popular applications of TMDs is using them as photodetectors, gas sensors, moisture sensors, and biosensors [118–124]. Among members of this family, sulphides and selenides of Mo and W are the most studied with several investigations on tuning their properties through chemical functionalisation or pairing them with other 2D materials, such as graphene, through van der Waal forces to form what is known as 2D heterostructures [124]. These hybrids possess very specific morphological and physical properties which make them promising candidates for various applications, such as sensing H_2_O_2_ or biomolecules like DNA, and tumours [125–127].

A recently discovered transition metals-based 2D family, which has grown rapidly, is transition metal (mostly groups 13 and 14) carbides and carbonitrides, known as MXenes. More than 70 types of MXenes have been synthesised and reported until now [128]. Ti_3_C_2_ was the first member of this group to be discovered, which was synthesised by exfoliation of Ti_3_AlC_2_ in 2011 [129] and opened a new door to the development of these 2D materials.

There are various methods suggested for synthesizing different MXenes. However, most of them follow the basic idea of developing a bulk MAX with structure of interest and then removing M–A bonds with the help of fluorinated agents like HF [130]. However, stirring or sonicating in the presence of KF, NaF, and LiF at high temperatures (550 °C) or other agents such as NH_4_HF_2_ [131] or LiF/HCl [132] as a replacement of HF produces the same results [133].

The most developed application of MXenes that remains in its first principals is their use in modern sensor development. Among such sensors are ultrathin dopamine sensors with an acceptable detection limit of about 100 nM [134], and flexible piezoresistive sensors with a fast response of about 30 ms [135]. Also, MXenes show advantageous applications as biosensors while coupling with other nanoparticles [136].

### Metal Oxides (MOs) and Metal–Organic Frameworks (MOFs)

2D MOFs are 2D and 3D solids mainly produced through self-assembly of metal ions and organic units of interest [137]. 2D MOF layers with similar dimension-related properties to other 2D frameworks such as large surface areas, high intrinsic porosities and abundant accessible active sites could be an attractive option for use in various functional sensory devices. MOFs have shown unique competitive advantages over other inorganic peers when applied in constructions of flexible or transparent devices [138–141].

2D MOFs can be synthesised through physical or chemical exfoliation of their bulk layered MOFs to form single-layer or few-layer MOFs [142–146]. Bottom-up methods such as the coordination interaction between organic precursors and metal ions components have also been used to prepare 2D MOFs. An important factor in this method is to adjust the growth rates of different crystal facets by selectively restricting the growth along the vertical direction, but allowing lateral growth in two dimensions [139, 147–152]. Other than high specific surface areas, the ordered porosity and abundant redox active sites in MOFs promise a superior adsorption–desorption characteristic towards gas, ions, and organic species [153–155]. MOFs, comprising light-harvesting chromophore bridging linkers or photoactive lanthanide ions (Ln^3+^), usually display luminescent nature under ultraviolet or visible excitation; the close interaction between fluorescent MOFs and analyte can change fluorescence behaviours of MOFs which provides an opportunity for the development of fluorescent sensing platforms to detect various analyte species [156–160].

MOs have been shown to be suitable candidates for chemical and bio-sensors. Nanotubes, nanoparticles, and other nanostructures of oxides of zinc, iron, cerium, tin, zirconium, titanium, and magnesium have shown ability to detect various molecules in solutions with high accuracy [161]. 2D MOs possess exceptional optical and electrical properties and high surface reaction activity which, along with their strong adsorption ability, makes them promising candidates for sensory applications [162]. Moreover, a large group of layered transition metal oxide compounds have the cation-exchange capability which could lead to the development of electrochemical sensory applications.

Individual 2D MOs, such as MnO_2_ [163] and ZnO [164], which have been used for detecting analytes (such as H_2_O_2_), can be fixed on support or be used in suspended form for sensing purposes [165]. Considering the fact that lower concentration of MOs can be applied for coating another base material, it seems to be a more logical option to develop sensors compared to employing MOs as the only compartment of a sensor responsible for both sensing and transducing.

Looking at the applications of MO-based sensors in forensic and industrial studies, we find a range of sensitive alcohol and drug detectors based on these 2D sheets, such as ultrathin WO_3_ layers which present an acceptable sensitivity for alcohols and are able to detect very low concentrations in a wide linear range [166]. ZnO nanosheets also function well as ethanol sensors at different temperatures [167]; before them, mostly one-dimensional MO nanoparticles were known for their alcohol detection properties [168–172]. The study of alcohol sensing applications of WO_3_ shows that by increasing the number of carbons, the sensitivity and response of sensor decrease; however, the recovery time for smaller alcohols was higher than their response time, which could also be related to slow evaporation rate of larger alcohols [166]. Porous ZnO sensors show various sensing behaviour against gases in different temperatures; they are able to detect chlorobenzene at relatively low operating temperatures (150 °C < *T* < 250 °C) and detect ethanol at relatively higher temperatures (250 °C < *T* < 450 °C). MO-based sensors have the potential to be highly affected by temperature and the weight of analyte molecules [167].

### *h*-Boron Nitride (*h*-BN)

Boron nitride consists of equal numbers of boron and nitrogen atoms. 2D hexagonal boron nitride (*h*-BN) is one of main stable BN allotropes and is actually an isomorph of graphene and is also known as “white graphene” [173]. It is an electrical insulator with an ultra-flat surface, highly stable with a honeycomb *sp*^2^-hybrid layer structure, while sub-lattices are occupied by equal numbers of boron and nitrogen atoms alternatingly [174]. The first isolation and experimental study of 2D *h*-BN were reported in 2008 [175].

Stable BN allotropes (2D *h*-BN and 3D *c*-BN) have been a studied for a long time because of their low density, high thermal conductivity, electrical insulation, superb oxidation resistance, excellent inertness, and low friction coefficient. 2D *h*-BN nanostructures possess a unique combination of these advantageous properties, which promotes their usage in various applications such as their use as dielectric substrates in graphene electronic devices, composite fillers, thermally robust catalytic and sensing substrates, highly durable field emitters, hydrophobic films, and so on [176–180]. At room temperature, the thermal conductivity of *h*-BN is up to 400 Wm^−1^ K^−1^, which is higher than the majority of metals and ceramic materials [181]. In addition, 2D *h*-BN is transparent in infrared and visible light and has no absorption in the visible range, but has absorption spectroscopy in the ultraviolet region (a strong peak around 251 nm) and a good photoluminescence property [182–184]. The shifts in ultraviolet, or emission peaks of *h*-BN or its derivatives in the presence of various analytes could be examined for sensory studies.

Various methods have been used to synthesise *h*-BN nanostructures, similar to the well-known techniques utilised for the growth of other 2D materials, including chemical vapour deposition, chemical exfoliation, mechanical cleavage, solid-state reactions, substitution reactions, high-energy electron irradiation, and unzipping nanotubes [178, 185–188]. CVD [189] on metallic surfaces such as Pt(111), Ru(001), and Ni(111) is one of the methods of preparation of layered *h*-BN by a chemical-solution-derived process. Mechanical exfoliation [175] and liquid exfoliation [190] are other supported and confirmed *h*-BN synthesised methods, where both are almost similar, but the latter one occurs through a vigorous stirring in a solution like DMF.

*h*-BN has also been used for various sensory applications, including the use of a composite of *h*-BN with graphene quantum dots as an electrochemical sensor for detecting serotonin in 10^−13^ M concentrations in urine samples [191]. In addition, another study on the properties of *h*-BN proved that the atomic layers of boron nitride could work as a resistance-based gas sensor and the electrical resistance of the surface was changed as it underwent reduction with tested CH_4_ gas [192].

## 2D Alcohol Sensors and Biosensors

**Fig. 3:**
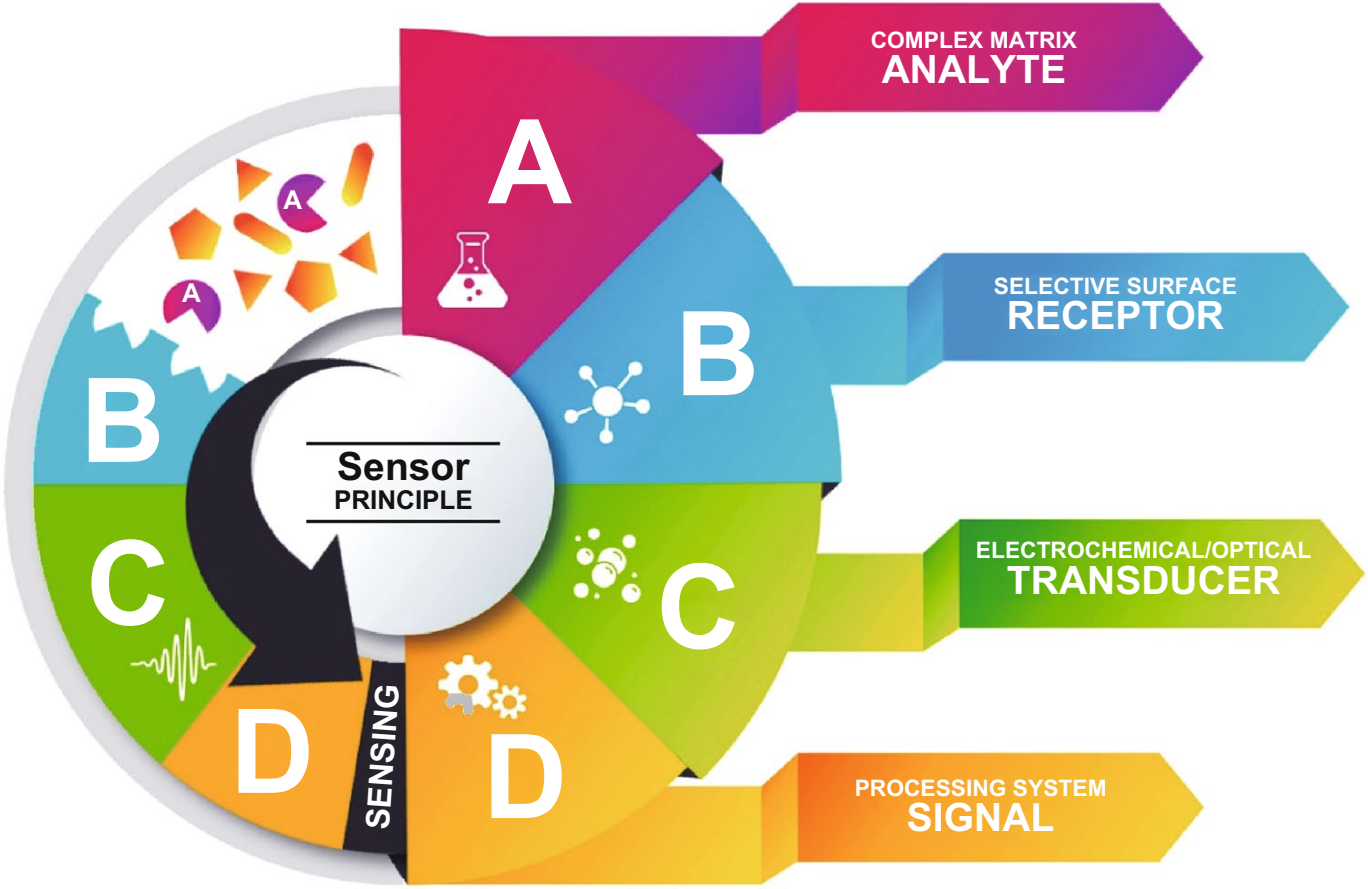
General scheme of a sensor. The interaction of an analyte with the selective receptor, coupled to a transducer, transfers a measurable signal to processing systems

The general structure of any chemical sensor (optical/electrochemical) is illustrated in Fig. 3. Chemical sensors contain two basic components connected in series: a chemical recognition system (receptor) and a physico-chemical transducer. In some cases, sensors might use a third compartment to do the amplification process, known as the amplifier [193, 194].

In order to develop a marketable product, the sensor should offer simplicity, stability, and easy usage. Also, based on the applications of the final product, it needs to be modified in different ways; for example, field sensors require portability, while pharmaceutical and medical studies ask for implantable probes for in vivo monitoring of analytes. Miniaturisation is another important factor, which reduces sample size required for analysis and also provides the possibility of packing multiple sensors in order to analyse several analytes at the same time, such as searching for different recreational drugs in one drop of blood. The nature of 2D nanomaterials provides the bed of miniaturised and portable sensors, while their size also allows in vivo studies as well.

2D materials can be used in sensors for four purposes: as a supporting agent that amplifies or facilitates the sensing; a transducer; a receptor; or both receptor and transducer at the same time. Working as a “supporting agent” means using them for other purposes that develop the sensing mechanism, but they are not a part of the main sensor, like using them as a conductive additive to the electrode or as an electrolyte solution to improve the sensor response. A transducer is responsible for transferring signals from the receptor to the signal processing system. Highly conductive 2D materials, such as graphene, are identical materials for the transducer and can decrease the response time of the sensor. The receptor is the main part of a sensor that directly deals with the analyte and selectivity, accuracy, and precision of a sensor directly related to the receptors.

**Fig. 4:**
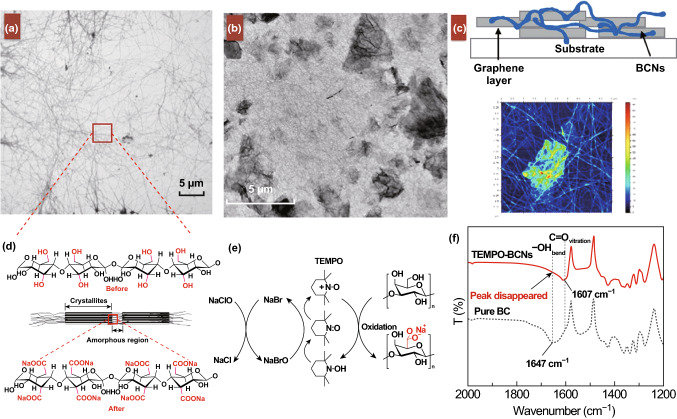
**a** TEM image of BCNs derived from BC gels. **b** TEM image of a graphene-BCN membrane. **c** AFM image of the structure of the graphene-BCN sensor. **d** Molecular structure of BCNs before and after modification with TEMPO. **e** Chemical reaction on BCNs caused by TEMPO-modification. **f** FTIR spectra of pristine BCNs and modified BCNs. Adapted with permission from Ref. [197]. Copyright 2017 Nature

Many alcohol sensing systems (examples listed in Table 1) are electrochemical sensors, which are made of a 2D material coupled with another compartment, such as a MO nanoparticle, for selective detection. The fact that 2D sensors could bring astonishing detection limits along with high selectivity in coupled forms could promise a bright future ahead of them. Reduced graphene oxide (rGO) and its derivative are at the forefront of 2D materials for sensor applications due to many factors such as availability, well-known chemistry, and the ability to tune the properties further by attaching new molecules to the basal plane. Analytes, such as buprenorphine, heroin, morphine, and noscapine [195, 196], can be detected with GO or rGO without any further modifications down to a detection limit of 0.2 μM. However, for small molecules like alcohols, especially in extreme situations such as high temperatures or high/low pH, graphene alone cannot detect low concentrations with reasonable sensitivity. The sensitivity of the graphene-based electrode can be enhanced by either attaching functional groups to the graphene basal plane or by mixing graphene with other organic or inorganic materials to form hybrids. Several wet chemical methods, as well as various functional groups, have been used to modify the graphene properties and improve the interaction at the surface–electrolyte interface (Fig. 4).

**Table 1:** Analytical parameters of 2D-based alcohol sensors and biosensors, sorted by LoD and type

Nos.	Analyte detected	Sensor type	Sensing materials	Limit of detection	Sensing matrix	References
1	Alcohols	Electrochemical sensors	Gr-Ns/BCNs	10%	Liquid and gas	[197]
2	Ethanol and propanol		PVA/CuO/Gr-NPls	1800 ppm	Gas	[198]
3	Ethanol, methanol, and isopropanol		rGO arrays	1500 ppm	Gas	[199]
4	Methanol		rGO/FL-NiO	100 ppm	Gas	[200]
5	Ethanol		Co_3_O_4_-HHMSs	100 ppm	Gas	[201]
6	Ethanol, ammonia, methanol, and acetone		TiO_2_/GO	100 ppm	Gas	[202]
7	Methanol		ZnCo_2_O_4_	100 ppm	Gas	[203]
8	Ethanol		SnO_2_	100 ppm	Gas	[204]
9	Ethanol		Au-NPs/SnO_2_	70.2 ppm	Gas	[205]
10	Ethanol		GO/Melamine	70 ppm	Gas	[206]
11	Alcohols		TiO_2_/MoS_2_	50 ppm	Gas	[207]
12	Ethanol		SnO_2_/MoS_2_	50 ppm	Gas	[208]
13	Ethanol		ZnO/MoS_2_	50 ppm	Gas	[209]
14	Ethanol		fSGO electrolyte film	25 ppm	Gas	[210]
15	Ethanol		ZnO/GO	10 ppm	Gas	[211]
16	Ethanol and NADH		Au–AgNPs/P(Cys)/rGO	5 ppm	Liquid	[21]
17	Ethanol		SnO_2_	5 ppm	Gas	[212]
18	Ethanol		α-Fe_2_O_3_/MoS_2_	1 ppm	Gas	[213]
19	Ethanol		Co_3_O_4_	0.2 ppm	Gas	[214]
20	Ethanol, acetone, and ammonia		Ti_3_C_2_T_*x*_	100 ppb	Gas	[215]
21	Ethanol and acetone		ZnSnO_3_	50 ppb	Gas	[216]
22	Ethanol, methanol, acetone, and ammonia		Ti_3_C_2_T_*x*_	34 ppb	Gas	[217]
23	Ethanol		*h*-BN	NA	Gas	[218]
24	Ethanol		Triphenylene frameworks/G	NA	Gas	[219]
25	Ammonia, ethanol, methanol, etc.		WO_3_/WS_2_	NA	Gas	[220]
26	Ethanol and methanol	Optical sensors	NaF/G	NA	Liquid	[102]
27	Alcohols and ketones		Au–rGO/SnO_2_/ZnO NWs	11 ppm	Gas	[221]
28	Ethanol		Ag/TiO_2_	5 ppm	Gas	[222]
29	Ethanol, methanol, and isopropanol		GO-BPOF	9–14 ΔRIU*	Liquid	[104]
30	Alcohols		MoS_2_	4.8 × 10^−6^ RIU*	Liquid	[223]

*RI refractive index—more details in the reference

Jiang et al. [210] attached sulphonyl groups to the surface of GO to increase its sensitivity and selectivity towards ethanol. Sulphonyl functionalisation of graphene oxide was accomplished with the help of 3-mercaptopropyl trimethoxysilane and then oxidation of its thiol group, which will finally form dark brown films. The sulphonic treated graphene oxide, or better rGO, was claimed to offer an increase in the conductivity and thermal stability of the sensor, while free-standing sulphonic acid-functionalised graphene oxide (fSGO) offers high selectivity for ethanol, compared to other chemicals like acetone. The sensor presented fast response time (50 s) and low detection limit (25 ppm) and offers high proton conductivity. Furthermore, humidity tests showed that by increasing the humidity, the resistance of the sensor decreases, which resulted in an increase in the response. The structure of the sensor was based on an alcohol fuel cell, which can be used in the construction of portable breathalysers. Despite the fact that graphene oxide offers thermal stability [224] up to 300–400 °C where it loses its oxygen functional groups [82], but it is poorly conductive [225] and there is a chance that graphene oxide actually reduced during the synthesis or functionalisation.

It has been suggested that the catalytic ability of noble metal nanoparticles (NMNP) such as Au [205, 221], Ag [222], or their bimetallic combination [21] could improve the sensitivity of the sensors towards alcohols. A biosensor has been recently fabricated from metal alloys, amino acids, and rGO and applied to study alcoholic and non-alcoholic beverages. The recovery values of this sensor demonstrated a good accuracy for detecting ethanol [21] as a result of this combination. The modified glassy carbon electrode (GCE/Au–AgNPs/P(L-Cys)-rGO) was prepared in three steps; firstly, gold and silver nanoparticles co-electrodeposited on the surface of the electrode. Afterwards, electropolymerisation technique was used to add a layer of poly(l-cysteine) coating. The electrochemically reduced graphene oxide film was electrodeposited onto the surface of the GCE/Au–AgNPs/P(L-Cys) electrodes via electroreduction of GO by cyclic voltammetry (10 cycles between +0.60 and −1.5 V at a scan rate of 25 mV s^−1^). The biosensor was developed through enzyme immobilisation (addition of enzyme solution containing alcohol dehydrogenases) and spreading a 2.0 µL nafion solution on the surface of the electrode. The developed biosensor was able to selectively detect both ethanol (LoD 5.0 µM) and nicotinamide adenine dinucleotide (LoD 9.0 nM) [21].

Another sensors for alcohol detection can be produced from bacterial cellulose nanofibres (BCNs) and graphene [197]. Extracted BCNs are reported to be highly crystalline (up to 90%) with ultrafine (2–50 nm) fibres, which offers high flexibility, high tensile strength, and moduli; their natural water absorbing characteristics lead to formation of porous foams after water removal [226, 227]. Thin films were fabricated under direct pressure over a gold electrode through vacuum-filtration and lamination technique. The whole system was fabricated over a plastic substrate (0.005, Clear Dura-Lar), and in order to increase the adhesion between the electrode and substrate, Ti was added to the gold electrode. The sensor offers reasonable sensitivity for both liquid and gas analytes which was suggested to be the result of the absorption characteristics of BCN. Different thicknesses were tested and 225 nm reported as the best thickness with optimum sensitivity. Despite the sensor’s good response to ethanol, its selectivity and response to other similar chemicals were not tested.

Thangamani et al. [198] mixed polyvinyl alcohol (PVA) and CuO nanoparticles with 6–8 nm thickness graphene nanoplatelets to fabricate a thin film using a colloidal blending method. PVA is reported to offer gel and film forming abilities, and also good porosity [228] Water solubility of PVA turns it into a popular organic additive to develop hybrid films [229–231]. Different ratios of CuO to graphene were tested, and the thickness of the film was in the range of 60–70 µm. The sensing properties of the film were investigated by recording the changes of the films electric resistance after exposure to different alcohol vapours in a controlled atmosphere test chamber. The change of the film resistance was attributed to the absorption/desorption of oxygen species and the subsequent reaction between oxygen species and the alcohol vapour molecules on the surface of the nanocomposites film. The reaction of the oxygen species (either O^−2^_(ads)_ and/or O^−^_(ads)_) with the alcohol generated electrons on the composite surface that changes the resistivity of the film. It was reported that by increasing the graphene loading, the sensitivity of the sensor increased due to the improvement in the film conductivity. However, above certain loading levels (0.5 wt%), the sensing behaviour declined due to the filler concentration being above the percolation limit. The sensor shows more sensitivity for propanol than ethanol. Also, the temperature had a considerable effect on the sensitivity due to the change of the absorption of oxygen species from physisorption type into a chemisorption type, which usually has stronger bonds with the substrate and increases the resistance of the film.

**Fig. 5:**
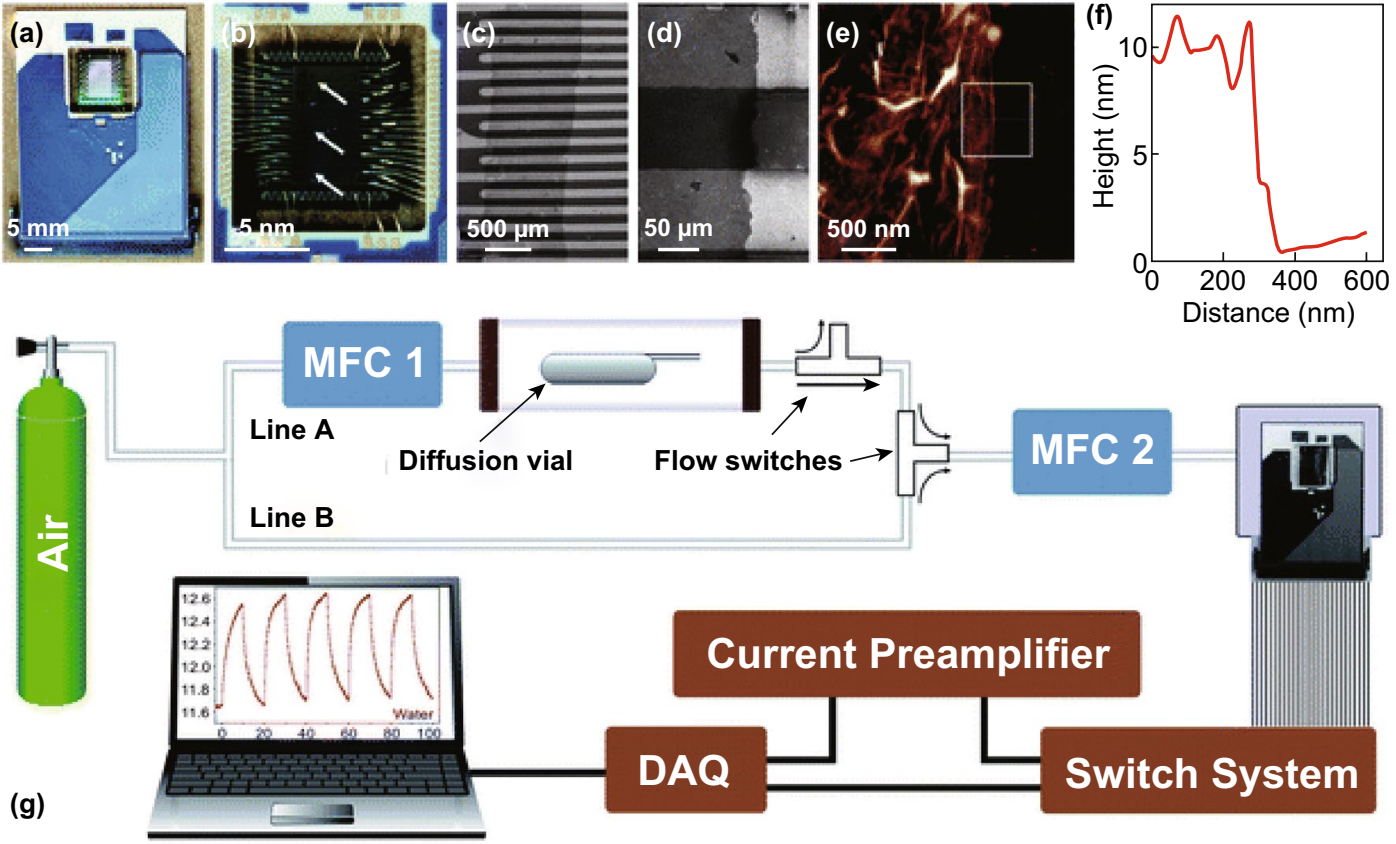
**a** rGO-based multisensor array. **b–e** SEM and AFM images of the chip. **f** A height profile of the film. **g** Schematic of the experimental set-up for sensor measurements. Adapted with permission from Ref. [199]. Copyright 2013 Royal Society of Chemistry

In order to increase the selectivity of rGO sensors, Lipatov et al. fabricated a large array of rGO devices by drop casting GO from a dilute suspension on KAMINA multisensor chip followed by gentle annealing at 150 °C under vacuum. A thin film of less than 10 nm thickness (approximately 10 layers of graphene) covered 39 Pt electrodes of the chips (100–3000 µm^2^ each) and the gap between them (~ 90 µm), forming 38 devices (Fig. 5). The sensing properties of the integrated array devices were measured by recording the change of the resistance during the exposure to 20 sccm flow of synthetic air with different concentrations of alcohol vapours. The resistance of the rGO device was found to increase with the time of exposure to alcohol gases, but then recovered the original conductivity when purged with dry air. The increase in resistance is quick at the first stage of alcohol exposure due to the fast kinetics of molecules adsorption at the low-energy binding sites, such as *sp*^2^ carbon domains. The rate of the adsorption then decreased as soon as the low-energy sites are occupied and the alcohol molecules are forced to bind to high-energy binding sites, such as defects and oxygen functional groups. Interestingly, the response of the rGO devices was different for different analytes. There were characteristic patterns for ethanol, methanol, isopropanol, and water that could be used to distinguish between different types of alcohol. It was also suggested that the selectivity of the rGO array devices might be enhanced if graphene is covalently functionalised with diazonium groups.

Lipatov’s suggested electronic-nose system was able to deal with the lack of sensitivity [199]. E-nose systems, other than selective chemical receptors, are strongly dependent on digital signal processing and pattern recognition procedures [232, 233]. The e-nose, calibrated with standards and signals from every individual sensor collected at the beginning, and the overlapped selectivity turns into a recognition algorithm which gives the sensing system the ability to detect the element of interest by comparison with the results of standard samples. The unique structure of graphene and its defects increase the chance of absorbing gas molecules when exposed to air, which changes the conductivity of this complex material and leads to detection. Every chemical tested (water, isopropanol, methanol, and ethanol) lead to losing the calibration of the e-nose by increasing the resistance, and it needs to purge with dry air to recover and respond accurately [199].

Zhang et al. [206] introduced porous melamine/GO sponge composite (GOMS) for selective ethanol sensing, and the sensor benefits from reasonably fast self-recovery. In order to form the composite, melamine sponge was first cut to size (3 cm^3^) and dipped into GO solution and pressed until all of the trapped air removed and freeze-dried. The immersing process tested for different concentrations of GO and the highest tested concentration (12 mg mL^−1^) offered a better conductive network and a larger response comparing to others. The sensor was tested among different gases, and it showed sensitivity towards acetone, methanol, and ethanol; though its sensitivity for ethanol was greatest, the sensor covered a wide linear range from 70 to 1670 ppm, which is higher than previously reported sensors such as graphene/ZnO with the linear range of 10–50 ppm [234] or MoS_2_/thiolat with a working range of 1–1000 ppm [235] for ethanol. Organic molecules incorporated with 2D materials such as graphene can also be used for sensing alcohols. Research on the applications of triphenylene frameworks in coordination with graphene layers conducted by Mathew and his team in 2017 [219] produced 16 different triphenylene derivatives. They successfully made a field effect transistor from a combination of graphene and one of their products, which showed a high and reversible response to ethanol vapour. The sensor was fabricated by placing a layer of graphene on Si wafer and then immobilising a certain developed triphenylene over it. Hydrogen bonding between ethanol and the amide functionality of applied triphenylene provides higher sensitivity towards ethanol compared to other tested gases (NH_3_, CO_2_, and H_2_), at room temperature.

Hierarchical flower-like (FL) NiO in a heterostructure with rGO was used to detect methanol, acetone, and ethanol under high humidity. Acetate salt of nickel was used to decorate graphene oxide with nickel oxide through a hydrothermal process. The final flower-like nanostructure was achieved by a thermal treatment step (1 h heating at 140 °C in an autoclave, followed by 3 h calcination at 250 °C of washed product), in which graphene oxide was reduced and nickel hydroxide was decomposed to give NiO. The specific surface area of the developed complex was measured by the Brunauer EmmeV Teller (BET) method and proved that addition of graphene improved the specific surface area of the sensor (5% rGO/FL-NiO and 10% rGO/FL-NiO offered a specific surface area of 165 and 160 m^2^ g^−1^, respectively). A paste of rGO/FL-NiO in isopropanol was casted over an alumina electrode substrate with gold arrays and dried at 250 °C. The interaction between the nano-flowers with methanol vapour affected the resistance of the sensor and leads to detection. The sensor showed the highest sensitivity against methanol, but could also sense acetone and ethanol as well [200]. Comparison of results suggested that addition of rGO could enhance the behaviour of the sensors to some extent, and authors noted that 5% rGO offered the best results; higher concentrations of rGO will wrap the Ni nanoparticles in a way that it actually blocks their active sites and weakens the response. However, it was suggested that higher amounts of rGO will decrease the working temperature of the sensor from 220 (for pure FL-NiO) to 180 °C.

A composite made of TiO_2_ and graphene is one of the few reported 2D sensors to offer room temperature alcohol detection, which uses the photocatalysis behaviour of TiO_2_ for this purpose. A homogenous solution GO with TiO_2_ nanoparticles was prepared in ethanol by sonicating for 2 h. A thin film of the nanocomposite was then prepared by drop casting 10 mg of the solution over a clean layer of polyimide. The composite sensor offered an enhanced normalised gas response for methanol, ethanol, and ammonia compared to pure GO layer; however, these nano-complexes suffer from short lifetime, and to deal with this issue, UV treatment of the sensor was performed that increased the lifetime of the detector up to 1 month and improved its resistance against water [202]. The UV-treated sample coloured differently, and this treatment also affected the behaviour of the sensor in a way that reduced the sensitivity of the sensor to different gasses and produced a higher response (normalised gas response) for ethanol than methanol.

Studies on different nano-morphologies of ZnO proved very good selectivity for ethanol as well, and the combinations of ZnO with GO, rGO, or other metal oxides lead to the development of various types of selective sensors [236–238]. Covering the surface of graphene oxide with rod-like nanoparticles of ZnO by a hydrothermal reaction is one example that leads to the fabrication of another sensor that responds selectively to relatively low concentrations of ethanol (10 ppm) [211], which offers a high response at different tested concentrations. The mechanism of sensing in ZnO nanoparticles is similar to other semiconductor gas sensors, where it is expected to adsorb some oxygen molecules when exposed to air, which would trap electrons from the conduction band of ZnO and produce adsorbed O_2_^−^, O^−^, or O^2−^. In an ethanol atmosphere, ethanol molecules could react with the adsorbed oxygen to form H_2_O and CO_2_ accompanied by the release of electrons and thus decreasing the resistance of the sensor [211, 239–241].

**Fig. 6:**
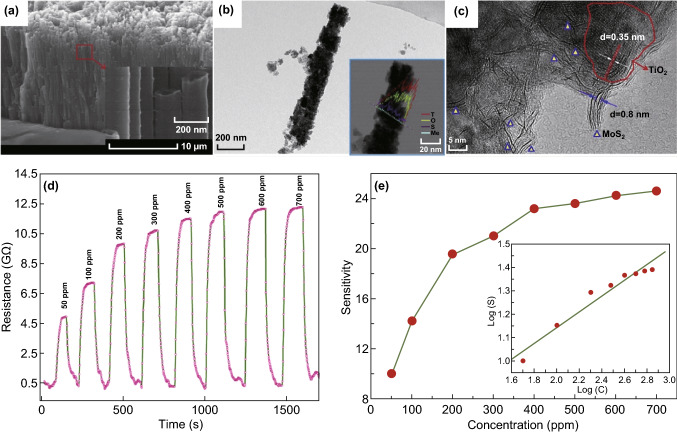
**a** SEM image of the TiO_2_ nanotubes, and the inset showing the opening top of the tubes, **b, c** HRTEM images of the MoS_2_–TiO_2_ composites, and the elemental distribution in the inset of **b**, **d** the response and recovery curves of the MoS_2_–TiO_2_ sensor to various ethanol concentrations from 50 to 700 ppm at 150 °C. **e** Sensitivity of the MoS_2_–TiO_2_ sensor at different concentrations of ethanol. Adapted with permission from Ref. [207]. Copyright 2016 Elsevier

A further study on TiO_2_ composites for alcohol sensing followed a different method, where 2D MoS_2_ was used to decorate TiO_2_ nanotubes [207]. The composite was produced through a hydrothermal reaction at 180 °C, and the final product showed reasonably good sensitivity for ethanol (14.2). The highest response appears at 150 °C increasing the temperature caused the sensitivity of the sensor to decrease; however, at 300 °C, the sensitivity increased again, but the response was not as high as at 150 °C. The sensitivity of the composite was reported to be 11 times more than TiO_2_ nanotube, yet the sensor suffers from lengthy response and recovery times (Fig. 6). Despite the similarities of various semiconductors’ sensing mechanism, which mainly comes from their interactions with oxygen and changing the resistance, they are not exactly the same. Non-decorated TiO_2_ nanotubes are *n*-type semiconductors, and after exposure to alcohols, electrons (which come from absorbed oxygen) are released back to the conduction band of TiO_2_ and finally decrease the resistance. However, TiO_2_ nanotube is covered with a *p*-type semiconductor (MoS_2_), the alcohols replace the absorbed oxygen and release free electrons to neutralise the holes in the *p*-type semiconductor, which results in increased resistance. TiO_2_ showed selective behaviour for certain alcohols in its various nano-morphologies in both high and low temperatures, and it is expected that other complex of this semiconductor with other 2D nanomaterials offers promising applications for alcohol sensing.

It can be seen that among the 2D alcohol sensors presented in Table 1, MXene-based sensors are performing very well with LoD at ppb levels which even surpassed many modified 2Ds, without forming any secondary complexes or adding modifications. Another important characteristic of Ti_3_C_2_T_*x*_ sensors is their ability to sense gases at room temperature [217] similar to TiO_2_, yet they have a longer service life. Although MXene sensors suffer from low selectivity and they may react with other gases including ammonia and acetone, they exhibit selectivity towards hydrogen-bonding gases over acidic gases [215]. This is probably due to the several functional groups on the MXenes surface and applying other transition metals, or further modifications could improve their selectivity. Density functional theory (DFT) investigations on the sensing mechanism of Ti_3_C_2_T_*x*_ and its behaviour against different gases, Ti_3_C_2_(OH)_2_ displayed a strong binding energy potential. Also, hydroxyl-group terminated MXenes displayed stronger gas adsorption compared to oxygen-group terminated MXenes. Therefore, it is assumed that the outstanding gas adsorption properties of the hydroxyl groups on Ti_3_C_2_T_*x*_ largely contribute to the high sensitivity observed in experimental studies [215]. That could justify the lack of selectivity of Ti_3_C_2_T_*x*_ against tested gases such as ethanol, methanol, etc., which also have been reported as electron donor gases [217, 242].

It should be noted here that while Ti_3_C_2_T_*x*_ was prepared by the same method in Refs. [194, 195], through etching Al from bulk Ti_3_AlC_2_ powders using LiF and HCl, but the fabrication of electrode was different. Lee et al. [217] used a drop casting method over a flexible polyimide film, while Kim et al. [215] used vacuum filtration method to produce free standing membrane of Ti_3_C_2_T_*x*_, which then transferred on thin SiO_2_ wafers. In the latter study, for better comparison of MXenes sensing abilities, thin layer of black phosphorous, MoS_2_, and rGO were also prepared and casted with the same method, where first subjected to vacuum filtration through a porous anodised aluminium oxide membrane (AAO) filter, and then, films separated from filter by dissolving alumina membrane with a NaOH aqueous solution before placing on a SiO_2_ wafer and drying. The comparison selectivity results for 100 ppm of different analytes showed that BP and MoS_2_ casted with this method showed the highest response against ammonia, while Ti_3_C_2_T_*x*_ was more responsive to ethanol and acetone. Also, electrical noise of each sensor was tested by measuring the resistance during N_2_ introduction, where BP showed the highest and Ti_3_C_2_T_*x*_ displayed the lowest noise level [215].

**Fig. 7:**
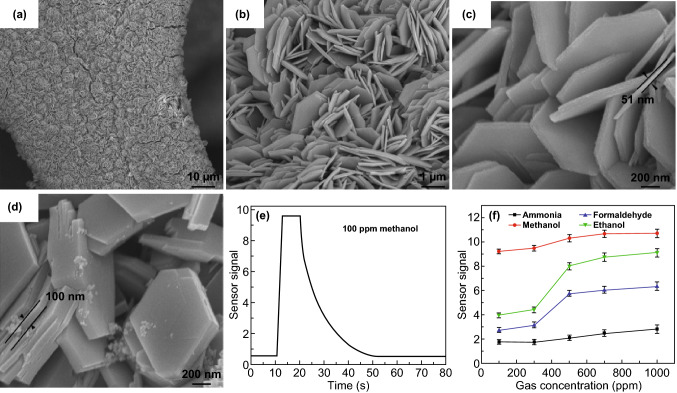
**a**–**c** SEM images at different magnification of hexagonal ZnCo_2_O_4_ grown on Ni foam using 0.2 mmol urea, **d** SEM image showed the increase in the sheets thickness with increasing the urea content, **e** sensor signal–time curve showing the fast response and slow recovery of the sensor, **f** the effect of the concentration on the sensor signal for various gases. Adapted with permission from Ref. [203]. Copyright 2018 Elsevier

In general, adding metal oxide nanoparticles to the active materials increases the selectivity of the sensors towards alcohol. The morphology, structures, and the interaction with other components in the composites play a crucial role in the performance of metal-oxides-enhanced 2D sensors. By comparing 2D nanomaterials decorated with nano-flower, hallow-spheres or rod-like metal oxides, as listed in Table 1, ultrathin 2D morphologies like ZnSnO_3_ [216] and Co_3_O_4_ [214] offer the best sensitivity. In addition, 2D layers offer faster response and recovery times, compared to their exact molecular formula formed other nano-morphologies. For example, 2D ZnSnO_3_ nanosheets [216] with a response and recovery time of 0.36 and 9 s, respectively, comparing to its nanorods (5 s) [243], hollow fibres (less than 10 s) [244], nanocubes (4 s) [245], and nanocages (2 s) [246], give the fastest response against ethanol. This could be attributed to the large specific surface area offered by 2D morphology, which facilitates the alcohol gas diffusion within the electrode. 2D ZnSnO_3_ was developed from 3D ZnSn(OH)_6_ cubes as a result of the hydrothermal reaction, where the cubes first decompose and then re-crystallised into porous nanosheets with a thickness of about 15–20 nm, by increasing the pressure and temperature. The developed 2D sensor was tested with various gases and showed the best response at 320 °C for ethanol (~ 4.5 *R*a/*R*g), but it was also sensitive to other gases and generated a detectable signal for gases like H_2_S, NH_3_, and acetone [216]. The same temperature effect is also reported for various MO-based alcohol sensors such as Cr_2_O_3_ [247], ZnCo_2_O_4_ [203], and Co_3_O_4_ [201], which shows the importance of operating temperature on the response semiconductor of gas sensors (Fig. 7).

Another example of 2D metal oxide alcohol sensor was the work of Yin et al. when they used ZnCo_2_O_4_ nanosheets to detect various gases [203, 248–250]. The ZnCo_2_O_4_ nanosheets were grown over a small piece of Ni foam substrate through a hydrothermal process at 200 °C. The produced nanosheets have a hexagonal structure with a thickness of ca. 84 nm. It was reported that urea affected the thickness of the nanosheets in a way that adding extra 0.5 mmol leads to increasing the thickness of the nanoparticles to 100 nm, but their regular hexagonal sheet structure remained the same; yet, adding 2 mmol urea increased the thickness to 70 nm and leads to aggregation and stacking of nanosheets together. When used as a sensor for various gases at 400 °C, the sensor signals, measured as the ratio between the resistance before and after gas exposure, were 9, 7.5, 5.1, and 2.0 to methanol, ethanol, formaldehyde, and ammonia, respectively. These results indicated good selectivity towards methanol, especially at a concentration below 300 ppm and high temperature (400 °C). The sensor also responded to high concentrations of volatile ethanol. In addition, ZnCo_2_O_4_ nanosheets sensor exhibited short response (25 s) and recovery time (23 s) at 400 °C for 100 ppm methanol.

**Fig. 8:**
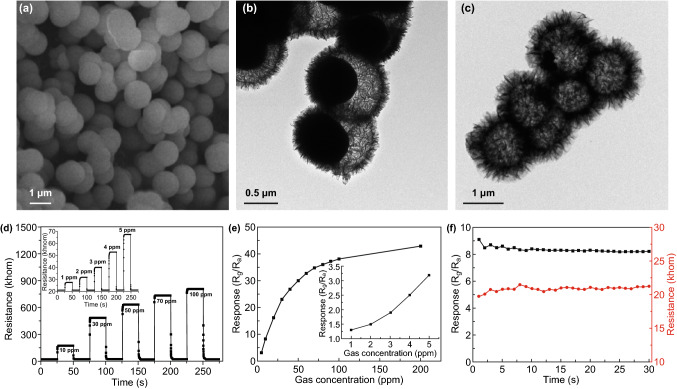
**a** SEM image of the Co_3_O_4_ microspheres, **b, c** TEM images of the hollow Co_3_O_4_ microspheres showing that the microspheres are composed of 2D nanosheets, **d** dynamic gas sensing transients of the sensors to ethanol with different concentrations at 220 °C. **e** Response of the sensor to various ethanol concentrations at 220 °C. **f** Long-term stability of the sensor to 30 ppm ethanol at 220 °C. Adapted with permission from Ref. [201]. Copyright 2017 Elsevier

Two-dimensional nanoparticles can also be used as the building blocks to construct larger porous microstructures. Tan and co-workers presented a new model of fabricated semiconductor sensors, where they fabricated 3D microspheres from 2D (3 nm thick) nanosheets (Fig. 8) through a hydrothermal reaction [201]. In order to study the gas sensing abilities of the developed nanostructure, hollow and hollowed-out Co_3_O_4_ microspheres (Co_3_O_4_-HHMSs) powder is first dispersed in water, before casting on an aluminium tube; then, Au electrodes equipped with Pt wires fixed on both ends of the tube. Dried powder forms a layer with thickness of 50 µm on the tube, and Ni–Cr alloy coil is fixed inside the aluminium tube as a heater. The sensor showed a very fast response (0.1 s) and recovery times (0.7 s) and offered a good selectivity towards ethanol at 220 °C.

Growing the ultrathin Co_3_O_4_ nanosheets on carbon foam templates is another way of fabricating high surface area electrode for sensors. Here, the carbon foam provides a backbone that protects the electrode integrity and also provides a conductive substrate that facilitates the movement of any electron that might result from adsorption reaction. It was not a surprise that Co_3_O_4_ nanosheets on carbon foam offer a lower working temperature of about 100 °C [214]. The 2D Co_3_O_4_ *p*-type semiconductor and on N-doped carbon foam the sensor provides the detection limit of 0.2 ppm for ethanol, which is one of the lowest values offered by 2D based sensors to date. The synthesis started with heating the carbon foam in a diluted mixture of sulphuric acid and nitric acid for 6 h, then a mixture of dissolved cobalt(II) nitrate and glucose was added on the foam, and after each few drops the foam is dried and the procedure repeated for several times. Then, the foam is transferred to a Teflon-lined autoclave filled with a mixture of cobalt(II) nitrate salt, water, and hexamethylenetetramine and heated to 120 °C before annealed at 250 °C to develop mesoporous Co_3_O_4_ nanosheets/N-doped carbon foam composite. The resistance of the obtained composite changed in the presence of ethanol and showed a good selectivity; yet its lengthy and complicated synthesis along with the longer response (45 s) and recovery (140 s) times compared to other Co_3_O_4_-based sensors [251, 252] are some of the drawbacks of this sensor.

Noble metal nanoparticles (NMNPs) are considered good catalysts for the dissociation of molecular oxygen species at a relatively low temperature. Hence, it was suggested that NMNPs facilitate gas molecules adsorption on the surface of metal oxides easier, resulting in higher-performance metal oxide-based sensors [205]. Liu et al. synthesised hexagonal SnO_2_ nanosheets loaded with Au nanoparticles that showed excellent performance for sensing ethanol gas. Firstly, SnS_2_ was produced inside a Teflon-lined hydrothermal reactor after 20 h heating at 180 °C; then, it turned to SnO_2_ by 2 h heating at 500 °C in air. Then, Au NPs decorated the surface of developed SnO_2_ nanosheets by adding a mild base (NaBH_4_) to the stirring mixture of obtained SnO_2_, HAuCl_4_, and lysine. The produced structure composed of aggregates of SnO_2_ nanosheets with a pore diameter of 5–10 nm and high specific surface area (64.2 m^2^ g^−1^) over gold nanoparticles [253]. The SnO_2_/Au nanocomposite showed maximum gas response at 260 °C. The ethanol gas response was almost 3.5 times more when SnO_2_ nanosheets were decorated with Au, proving the crucial role of the noble metal NPs on improving the sensitivity of the sensor [205]. The effect of Au addition was less pronounced when sensing other gases, acetone, and ammonia, probably because the sensitivity towards these gases was low. Therefore, SnO_2_/Au nanocomposite sensor was selective towards ethanol which agrees with other SnO_2_ nanosheets sensor studies [204].

Another porous nanosheets of SnO_2_ sensor for ethanol detection were recently developed by Zhao et al. [212]. GO was used as a template for SnO_2_ nanosheets growth in a hydrothermal process, and oxides functional groups at the surface helped to direct the development of the porous structure. It was found that GO was also controlling the size and thickness of SnO_2_ nanosheets. By increasing the amount of graphene oxide, the size of SnO_2_ nanosheets gets smaller from several micrometers to a few tens of nanometres. After the end of hydrothermal treatment, GO was removed by annealing at 500 °C in air, giving a hierarchical porous microstructure with the porous size in the range from 2 to 20 nm and specific surface area as high as 80.1 m^−2^ g^−1^. To form the sensor electrode, the produced SnO_2_ nanosheets are dispersed in ethanol and deposited on oxidised Si substrate with Cr/Au electrode arrays and heated at 400 °C for 2 h. The electrode was then heated at 300 °C for a week to improve its stability. The sensing measurements were taken at temperatures between 150 and 300 °C, while the relative humidity was around 40%. The hierarchical porous nanosheets-based sensor exhibits a high response value of about 71, based on the resistance ratio, for a wide range of ethanol concentrations (5–5000 ppm). It was also found that the response of the sensor improved by decreasing the SnO_2_ nanosheets, i.e. by increasing the GO content in the initial charge. The idea of using graphene sacrificial scaffolds could be used to synthesise other 2D materials nanostructures.

Yu et al. [221] tried to use advantages of noble metals, graphene, and metal oxide in one composite; they developed ZnO doped SnO_2_ nanowires and applied them over Au–rGO surface to sense alcohols and ketones by cataluminescence (CTL). The substrates of Au–graphene for both GO and rGO are prepared through similar methods where GO (or rGO) is refluxed in the presence of chloroauric acid and sodium citrate, and after turning the colour of the solution from black to purple, it is washed and dried at 80 °C. The substrate is coated with tungsten coil, and atmospheric pressure chemical vapour deposition (APCVD) was used to deposit SnO_2_–ZnO nanowires on it. By measuring the luminescent emission of electromagnetic radiation, which is produced during the catalytic oxidation reaction of gas molecules on the surface of solid catalyst, they were able to achieve a detection limit of 11 ppm for ethanol at 260 °C. It was reported that higher temperatures (> 260 °C) offer a stronger CTL response, but signal to noise ratio was decreased. The sensor also responded to ethanol when Au was removed (rGO–SnO_2_–ZnO) and without ZnO (solo SnO_2_–Au–rGO), but the intensity of the response was much higher in a composite made of all of them together. The sensor was responding to different types of alcohols and ketones, but not other organic solvents; the highest response was to ethanol and methanol, respectively.

Heterostructures of semiconductor 2D TMD with MO as another semiconductor showed enhanced sensing capability for alcohol detection, particularly when a *p*–*n* junction can be formed. Both MO and 2D TMD would have the same Fermi energy level at the interface, which results in a staggered band offset and a built-in internal electric field. When using the heterogamous structure in a sensor, the electron generated from the adsorption reaction can easily move across the interface and transfer to the conductive band. One example for ethanol detection used sensor based on *n*-type semiconductor α-Fe_2_O_3_ hollow microspheres on MoS_2_ nanosheets prepared by layer by layer self-assembly method [213]. Alpha-iron oxide and MoS_2_ were prepared separately using hydrothermal method followed by heat treatment. The electrode was then fabricated by casting five layers of MoS_2_ and α-Fe_2_O_3_ on the substrate of a printed circuit board with interdigital electrodes. Before casting the α-Fe_2_O_3_/MoS_2_, two layers of poly-(diallyldimethylammonium chloride) and poly-(sodium 4-styrenesulphonate) PDDA/PSS were deposited as precursor layers for substrate charge enhancement. The developed sensor was able to detect ethanol at concentrations as low as 1 ppm with high response. The response time was as short as 6 s and recovery time was 5 s, shorter than α-Fe_2_O_3_ or MoS_2_ devices. The enhancement of the sensing performance of the α-Fe_2_O_3_/MoS_2_ was attributed to the increase in the active sites for gas molecules adsorption, such as defects or oxygen vacancies, when α-Fe_2_O_3_ into MoS_2_ nanosheets.

Yan et al. prepared another 2D TMD–MO (SnO_2_–MoS_2_) composite sensor with *p*–*n* junction for ethanol detection. The composite was prepared by hydrothermal co-treatment of tin and molybdenum salts [208]. The sensor casted on an aluminium tube attached to gold electrodes, while supplied with a Ni/Cr heater inside the tube. The changes in the resistance of the sensor were used to measure the amount of ethanol in its surrounding atmosphere. A response as high as 120 (*R*_a_/*R*_g_) was obtained for ethanol at 280 °C when the heterostructure was used, compared to a response of about 40 (*R*_a_/*R*_g_) at an optimum temperature about 350 °C when SnO_2_ film was used. The SnO_2_–MoS_2_ composite sensors also showed good selectivity towards ethanol.

ZnO nanoparticles on MoS_2_ nanosheets were another composite with *p*–*n* heterojunctions that have been tested for ethanol detection. The composite was prepared using two hydrothermal steps to produce 2D flower-like structure consisting of MoS_2_ nanosheets decorated with 10 nm particles of ZnO [209]. Investigating the sensing properties of the nanocomposite at different temperatures showed that the response to ethanol reached a peak of about 42 at about 260 °C. The response at all temperatures was higher than that of ZnO nanoparticle, nanorods, and flower-like nanostructure, proving the role of the *p*–*n* junction on enhancing the performance of the sensor. The electrode showed good response at various concentration of ethanol in the range of 50–1000 ppm, unlike the ZnO electrode, which was less sensitive at low gas concentration. The ZnO–MoS_2_ electrode was more sensitive to ethanol than other tested gases, including methanol and ammonia, which makes the electrode suitable for selective detection. Jha et al. prepared composite of WO_3_/WS_2_ by sonicating a mixture of bulk oxides in isopropyl. The sensor fabricated from WO_3_/WS_2_ composites showed more selectivity towards ammonia and a lower response to small alcohols [220]. It should be mentioned here that there are some reports about WO_3_ changing its conductivity when the bulk particles are sonicated to the nano level, and therefore, the WO_3_/WS_2_ composite may not have enough *p*–*n* junctions to facilitate the electron transferee and work as active sites to attract oxygen from alcohol.

Fibre optics are well-known types of optical sensors and biosensors that apply optical fibres as transducers for light which showed a good application in sensor development for environmental, medical, and chemical purposes [254–256]. Studies on 2D materials, such as GO which showed low reflective indexes [257], give the idea that the low refractive index difference between refractive indexes of fibres core and layers of 2D materials could be used to develop an optical sensor, where using a different number of (2D material) layers could affect the sensitivity of the sensor [104]. This finally leads to using various 2D materials in the construction of fibre optics to detect different analytes such as humidity or NH_3_ [258–260]. Very recently, the application of 2D materials in the development of novel fibre optics for detection of alcohols in near IR region is reported [102, 223]; both sensors [102, 223] responded towards simple alcohols, yet the fluoride–graphene sensor showed a peak for ethanol at higher degrees (angle) comparing to methanol, while it is completely the opposite for the polymer–MoS_2_ sensor. The polymer–MoS_2_ sensor is actually made of a samarium doped chalcogenide (Se_95_Te_5_Sm_0.25_) as fibre core which covered with four layers namely a clad (perfluorinated polymer), Ag (42 nm thick), MoS_2_ (0.71 nm thick), and polythiophene (7 nm thick) from the core to outside. The detection mechanism is based on measuring the power loss occurred in the SPR sensing region; connected processing system shows this power loss as a sharp peak, and in the presence of alcohols, this peak shows a shift [223].

Laser sources of longer NIR wavelength lead to a better detection limit of the sensor. However, it decreases the sensitivity of the sensor in both mentioned sensors. A U-bent optical fibre sensor made of a polymethylmethacrylate core which is coated with GO and showed a sensory behaviour against solvents from simple alcohols to more complex organic solvents [104]. For coating the surface with GO, the hexamethylenedi-amine functionalised U-bent plastic optical fibre (BPOF) probes are simply dipped in different concentrations of GO solution (GO dispersed in water by sonication) and experience heat treatment for 24 h. Control probe (bare) and GO covered probes were coupled to a halogen light source from one side and a spectrometer from the other side to study the response of the developed sensor. It was suggested that by adding alcohols to the sensing environment, the absorbance of the sensor increased due to alcohols ability to effectively intercalate the GO layers and increasing the RI of the GO layer. This increase in RI around fibre core reduces the RI contrast and thus leads to improved absorbance response; however, alcohol might also react with the functionalisations and removes some of it to affect the RI contrast. The surface structure was scanned with Raman after various heating periods in the incubator, and the most uniform structure was obtained after 24 h heating. The highest tested coating concentration of GO (100 µL mg^−1^) presented the best sensitivity among all [104].

It has been suggested that few layered black phosphorus (BP) could be used for detecting alcohols like methanol with a limit of detection (LoD) of about 28 ppm [261]. However, black phosphorus, as opposed to phosphorene, is considered more of bulk material and its stability, especially against moisture, is low. Its application for sensing chemicals other than H_2_O seems to be far from implementation, especially for complex matrixes, so it is not discussed in this paper.

The ability of *h*-BN to detect small alcohols is not in question, whether in solo [218] or heterostructure form [262], but if there are more complex organic molecules in the matrix, such as petroleum gases or chloroform, it will be preferentially attracted to them over small alcohols like ethanol [263]. This attraction towards larger organic/bio-organic molecules along with the huge surface of 2D *h*-BN has even lead to the development of single-molecule detection systems to detect certain analytes like DNA [264]. Surface modification could change this trade and allow *h*-BN to work as a more selective sensor for small alcohols.

Despite available methods that can directly measure ethanol in various matrices such as body fluids or vapours, the lifetime of ethanol in the body is short and its metabolic pathway will rapidly distribute it through the body. Especially when it comes to medical, forensic, or even workplace alcohol consumption tests, some of the ethanol metabolites can be used to measure alcohol intake, indirectly. Ethyl glucuronide (EtG), ethyl sulphate (EtS), fatty acid ethyl esters (FAEEs), and phosphatidylethanol (PEth) are some of the main biomolecules and biomarkers which are currently used in the examination of alcohol drinking and in monitoring therapeutic procedures [265–267]. The level of these ethanol metabolites is mostly studied and measured through analysing different body fluids or tissues (hair, nail, meconium, blood, etc.) by various chromatography approaches, mainly GC–MS/MS and LC–MS/MS [5, 265, 266, 268–272]. Due to the complexity and high costs of these techniques, demand for easy to use, cheaper, and portable sensors is slowly rising, and to address this issue, nanosensors could come in use [273–275]. In a very recent study, ZnO was used in developing an electrochemical immunosensor [274]. Firstly, a thin layer of Au (60 nm thickness) is deposited on a flexible polyethylene terephthalate (PET) substrate through e-beam evaporation. Afterwards, 2D ZnO is separately prepared and then sonicated in the presence of Au–PET substrate, while the temperature is kept below 50 °C during sonication. Then, the developed composite is rinsed with water and dried under N_2_ before the addition of antibody. Finally, EtG antibody is immobilised on the sensor by its electrostatic interactions with ZnO. Added 2D ZnO actually worked as a glue which assists the binding of the antibody to the surface of the sensor without any need for an additional layer to make and maintain this link. The biosensor offers an LoD of 1 ng mL^−1^ and a linear range up to 100 µg mL^−1^ for ethyl glucuronide. Correspondingly, when it comes to forensic or pharmaceutical studies, working towards developing selective sensors to detect ethanol metabolites could be an interesting field of work and 2D materials for example graphene or metal oxides could offer many benefits from mechanical strength and flexibility to high conductivity and affordability for such sensors.

## A Comparison Between Nanosensors

Other than the examples of chemical sensors and conventional alcohol sensing methods which were discussed in the introduction of this paper, a lot of different nanosensors with various nanomorphologies have been developed for alcohol sensing purposes, including zero-dimensional nanoparticles (0D, such as clusters, spheres, and dots), one-dimensional nanomorphologies (1D, such as tubes, wires, rods, and fibres) and their combinations. Here, some of those alcohol nanosensors are discussed briefly (Table 2) to present a comparison with two-dimensional morphologies in terms of detection limits, sensitivity, and selectivity. The sensing mechanism is similar, regardless of changes in nanomorphology. In many cases, the sensing mechanism is based on the interaction between the reductive gases and the oxygen on the surface of sensors. This oxygen adoption on the surface of the sensor stops free electrons from the conduction band forming a space charge layer and eventually results in a higher resistance. The exposure to reductive gas molecules releases the trapped electrons back to the conduction band and increases carrier concentration which leads to a decrease in the resistances of sensors [276].

**Table 2:** Analytical parameters of alcohol nanosensors and biosensors, sorted by LoD and type

Nos.	Analyte detected	Sensing materials	Limit of detection	Sensing matrix	References
1	Ethanol and NADH	SWCNT-PBCP	0.1 mmol L^−1^	Liquid	[277]
*2*	*Ethanol and NADH*	*Au–AgNPs/P(Cys)/rGO*	*5* *ppm*	*Liquid*	[21]
3	Ethanol	PbS QDs/ZnO NRs	100 ppm	Gas	[278]
4	Ethanol	ZnO NRs/MWCNTs	5 ppm	Gas	[279]
*5*	*Ethanol*	*ZnO/MoS*_*2*_	*50* *ppm*	*Gas*	[209]
*6*	*Ethanol*	*ZnO/GO*	*10* *ppm*	*Gas*	[211]
7	Ethanol	α-Fe_2_O_3_ nanofibres	100 ppm	Gas	[280]
*8*	*Ethanol*	*α-Fe*_*2*_*O*_*3*_*/MoS*_*2*_	*1* *ppm*	*Gas*	[213]
9	Ethanol	Ag/TiO_2_ nanobelts	300 ppm	Gas	[281]
*10*	*Ethanol, ammonia, methanol, and acetone*	*TiO*_*2*_*/GO*	*100* *ppm*	*Gas*	[202]
11	Ethanol	Ni-doped SnO_2_	0.6 nM	Gas	[282]
*12*	*Ethanol*	*SnO*_*2*_	*5* *ppm*	*Gas*	[212]

Rows that contain 2D sensors are showed in italic

Through comparison of several sensors with similar molecular structures that possess different morphologies, one can see 2D-based sensors are working better in terms of detection limits or response times. For example, the LoD of a 2D graphene-based sensor which is based on alcohol dehydrogenases [21] is 20 times better than the LoD of carbon nanotube-based 1D sensor which utilises the same enzyme for sensing ethanol [277]. Another example is α-Fe_2_O_3_ nanofibres prepared by an electrospinning process which showed a LoD of 100 ppm as well as a fast response and recovery of 3 and 5 s, respectively. A 2D-based sensor of α-Fe_2_O_3_ with MoS_2_ showed a LoD of 1 ppm for ethanol [213], which is 100 times greater than the 1D morphology (nanofibre) of similar chemical composition [280]. A composite made of PbS QDs and ZnO NRs developed a porous flower-like nanostructure which was able to sense ethanol gas at room temperature with a detection limit of 100 ppm [278].

Another room temperature ethanol gas sensor was developed from combination of two 1D nanoparticles, i.e. ZnO nanorods and multi-walled carbon nanotubes (MWCNTs). The linear range of the sensor was reported to be between 5 and 5000 ppm, while the response time of the sensor against 50 ppm ethanol was found to be about 7 s which was almost half of pure ZnO response (13 s) to the same amount of ethanol [279]. A sensor based on a 2D hybrid that contains ZnO and graphene was able to achieve a detection limit of 10 ppm, which is 10 times better than that of QDs and 5 times better than 1D based sensor [213]. 1D TiO_2_ nanobelts and Ag–TiO_2_ nanobelts were used to formulate an ethanol sensor; the response time and the sensitivity for different concentrations of ethanol from 5 to 500 ppm were tested, and they were weak below 300 ppm [281]. On the other hand, a sensor developed from 2D TiO_2_ offers much better sensitivity (100 ppm), yet the 1D sensors seem to work better in terms of selectivity [202]. Choi et al. conducted a systematic study using four different morphologies of Co_3_O_4_ as gas sensors [283]. Almost all of the structures responded selectively towards ethanol, compared to other tested gases (hydrogen and carbon dioxide). A comparison of agglomerated micro-size powders of Co_3_O_4_, its nanosheets, nanorods, and nanocubes showed that nanosheets had the strongest response to ethanol. Co_3_O_4_ nanosheet showed a response of 57.7 *R*_g_/*R*_a_ towards 100 ppm of ethanol, while nanorods, nanocubes, and micro-powder showed *R*_g_/*R*_a_ responses of 25.7, 24.7, and 5.5, respectively.

## Concluding Remarks and Outlook

The potential applications of ultrathin materials in sensing and biosensing are persuading increasing numbers of scholars to probe deeply into their properties and applications. 2D materials are promising elements of electrochemical and optical sensors. With the outstanding surface to mass ratio and unique physical and chemical properties, 2D materials are able to provide an extensive library of additional candidates for broad applications in developing sensors. In this review, we first discussed the chemical and physical characteristics of stable 2D materials with application in alcohol sensing and looked at some of their synthesis methods. Then, we briefly addressed the fundamentals of the sensors and how 2D materials could benefit the development of sensors and biosensors. Finally, a comprehensive review of selected examples of 2D materials as highly sensitive sensors for industrial, forensic, pharmaceutical, and analytical study of alcohols was presented to the reader.

Graphene is still one of the most widely used 2D materials for general sensory uses, and it also works as a perfect substrate for coupling with other chemicals or biochemicals in order to amplify the selectivity of the sensor, but other 2D materials, as discussed in this paper, are proved to offer better properties such as improved LoD, different working temperature and so forth. One of the main challenges in the way of exploration of 2D materials’ properties, function, and applications, other than graphene, is the lack of facile, feasible, and reliable methods for large-scale preparation and sealing them. The second field to work on is finding functionalisation methods and developing methods to anchor selective receptors directly on the surface of 2D materials, which is still the matter in question even for well-established reduced graphene oxide layers. Also, as proved, a combination of two different 2D materials leads to optimised properties and searching to find other possible combinations for various uses could be another field of study for the future. Using GO-based solid-state electrolyte films to replace conventional liquid acids was an example of how 2D materials could work towards evolving old technologies. Promising applications of MOFs in sensing alcohols could be merged with the benefits of 2D morphologies, to achieve characteristics such as larger surface area to achieve lower detection limits. Deeper investigations on the 2D sensors’ selectivity, multiplexity, durability, and reusability can help better understanding of their nature and help scholars to focus more on composites which may offer better application for industrial developments. Large surface of thin materials improves the interactions between sensor and matrix, yet in some of the discussed sensors, their functional groups and sensing mechanisms play a more important role than their thickness to sense alcohols. Doping metal oxide nanosensors with other metals can drastically improve their sensing characteristics in terms of selectivity and sensitivity as it can be seen for SnO_2_ in Table 2. One of the main things that we understand from analysis of the literature is that real samples, especially biological samples, such as blood, are not well studied yet and most of the real samples were artificial, laboratory-made samples like a mixture of gases in a certain range; so working towards developing an alcohol sensor to work in relatively lower temperatures with application in bio-analytical studies could be an interesting field of study for 2D sensors. We hope this review will guide future developments that can enhance new sensory technologies which not only respond to industrial demands, but also protect our environment, health, and safety.
